# Translating Mortality Hazard Ratios into Remaining-Lifespan Measures in Older Adults Using Japanese Complete Life Tables: A Deterministic Reference-Table Study

**DOI:** 10.3390/geriatrics11050116

**Published:** 2026-08-31

**Authors:** Takashi Shibuya, Nobuo Kutsuna, Tomoya Ohida, Miwa Nakamura, Hanako Kinugasa

**Affiliations:** 1 Corporate Strategy Division, Fukujukai Medical Corporation, Tokyo 123-0851, Japan; takashi.shibuya@fukujukaigr.or.jp; 2 Department of Neurosurgery, Fukujukai Adachi Tobu Hospital, Tokyo 121-0816, Japan; tomoya0905japan@gmail.com; 3 Department of Stress and Invasiveness Control, Toho University School of Medicine, Tokyo 153-8515, Japan; 4 Department of Pharmacy, Fukujukai Adachi Tobu Hospital, Tokyo 121-0816, Japan; frommiwa.nkmr@gmail.com (M.N.); hanakumako87@yahoo.co.jp (H.K.)

**Keywords:** life table, hazard ratio, remaining lifespan, survival probability, older adults, geriatric epidemiology, Japan

## Abstract

**Background/Objectives:** Mortality hazard ratios (HRs) do not directly express remaining lifespan or fixed-time survival. We constructed Japanese age–sex–HR reference tables and examined uncertainty, time patterns, and age-based translation. **Methods:** Using the 23rd Complete Life Tables for Japan, 2020, we transformed annual death probabilities at index ages of 65–90 years with HRs of 1.00–4.00. Outcomes were median remaining lifespan; mean remaining years; 1-, 3-, 5-, and 10-year survival; and a baseline-equivalent age shift obtained by matching each transformed median to the HR 1.00 median curve. **Results:** At the age of 75 years, the median remaining lifespans at HRs of 1.00, 1.50, 2.00, 2.50, and 4.00 were 12.6, 10.1, 8.4, 7.3, and 5.1 years in men and 16.8, 14.3, 12.6, 11.3, and 8.8 years in women. An HR of 2.00 corresponded to baseline-equivalent age shifts of 5.8 and 4.8 years. A hypothetical HR interval of 1.50–2.50 translated to median ranges of 7.3–10.1 and 11.3–14.3 years. Relative to an HR of 2.00 throughout, a one-year front-loaded HR of 4.00 followed by an HR of 2.00 reduced median lifespan by 0.42 and 0.19 years but reduced 5-year survival by 3.8 and 1.9 percentage points. **Conclusions:** Life tables provide reproducible translations of HRs. Applying an external HR requires proportionality and transportability assumptions; the outputs are not validated patient-specific prognoses.

## 1. Introduction

Population ageing has increased the number of clinical decisions in which the expected time to benefit matters [1,2]. In older adults, the balance of benefits and burdens of cancer screening, preventive treatment, rehabilitation, treatment intensification, deprescribing, and advance care planning often depends on whether a person is likely to survive for a clinically relevant period. Prognostic indices can support these discussions, but many require participant-level variables, fitted regression coefficients, or web calculators that are not readily interpretable during routine consultation [3,4,5,6].

The hazard ratio (HR) remains one of the most common effect measures in survival analysis and is central to the Cox proportional hazards model [7]. Its interpretation nevertheless remains debated because of non-collapsibility, selection over follow-up, dependence on the proportional hazards assumption, and the gap between relative hazards and absolute survival probabilities [8,9,10]. Even when an HR is statistically valid within its source study, readers may still need to understand what an HR of 1.5, 2.0, or 3.0 implies for remaining years of life at a given age.

Life-table methods provide a direct route from age-specific mortality to survival probabilities and remaining years of life [11]. Proportional changes in mortality hazards have previously been combined with life tables for quantitative impact assessment and estimation of changes in life expectancy [12,13]. Age-based translations of mortality ratios, including effective-age and longevity measures, have also been developed as communication tools under assumptions about proportional hazards and age-related mortality patterns [14,15]. In Japan, complete life tables provide publicly available age- and sex-specific one-year probabilities of death [16]. These data allow specified HRs to be translated into absolute remaining-lifespan measures without presenting the result as a newly fitted patient-level prediction model.

The objective of this study was to construct a reproducible age–sex–HR reference grid for Japanese older adults. Specified mortality HR inputs were translated into median remaining lifespan, mean remaining years, fixed-time survival probabilities, and a baseline-equivalent age shift. We also quantified agreement with official remaining life expectancy, propagated an illustrative HR confidence interval through the transformation, and examined purely hypothetical time-varying HR sequences. The scenario analyses were disease-neutral and were not intended as predictions for any clinical condition.

## 2. Materials and Methods

### 2.1. Study Design

This was a deterministic life-table translation study. No participant-level data were used, no regression coefficients were estimated, and no calibration or discrimination analysis was attempted. The life-table baseline represented the Japanese general population in the 23rd Complete Life Tables for Japan, 2020 [16]. The intended output is a reference translation of specified mortality HR multipliers. The grid does not establish whether an HR reported in another study is transportable to the life-table baseline.

### 2.2. Life-Table Data

We extracted the age- and sex-specific one-year probability of death, denoted by $q_{x,s}$, from the national male and female tables of the 23rd Complete Life Tables for Japan, where x is attained age and s is sex [16]. The HR of 1.00 in this study represents the general-population life-table baseline. It does not represent a disease-free, frailty-free, cancer-free, or otherwise unexposed population. This distinction is central to the interpretation of the results because the general population already contains people with chronic diseases, disability, and high-risk conditions at their prevailing frequencies.

We also transcribed the official remaining life expectancy reported for the 12 age–sex index cells. The published death probabilities terminate at age 113 years in men and 114 years in women; to close the survival curve, we assigned a death probability of 1.0 at the next attained age. For the index ages studied, this terminal closure changed computed mean remaining years by less than 0.00001 years. Agreement between calculated mean remaining years at an HR of 1.00 and the official values was summarized by the mean absolute error, maximum absolute difference, mean absolute percentage error, and maximum absolute percentage error. Because the published one-year death probabilities and official remaining-life-expectancy values are reported to five and two decimal places, respectively, the agreement measures primarily reflect finite source precision and the trapezoidal integration step rather than participant-level model error.

### 2.3. Hazard Ratio Transformation

For each age and sex, we treated a specified HR as a multiplier of the annual life-table hazard. In discrete time, the transformed one-year probability of death was calculated as $q_{x,s}^{\prime }\left(h\right)=1-{\left(1-q_{x,s}\right)}^{h}$, where h is the HR and $q_{x,s}^{\prime }\left(h\right)$ is the transformed probability for age x and sex s. Conditional survival from index age a to integer residual time t was calculated as $S_{h}\left(a,t\right)=\prod_{k=0}^{t-1}\left(1-q_{a+k,s}^{\prime }\left(h\right)\right)$. Median remaining lifespan was defined as the first residual time at which survival reached 0.5, with within-year interpolation given by $S_{h}\left(a,n+u\right)=S_{h}\left(a,n\right){\left(1-q_{a+n,s}^{\prime }\left(h\right)\right)}^{u}$, where 0≤u<1. Mean remaining years were calculated by trapezoidal integration of the annual survival curve. For a time-dependent scenario, we replaced the single h by a sequence $h_{k}$ at residual year k.

The fixed-HR grid applies the same multiplier at every attained age and annual interval over the remaining-life trajectory. This is a proportional-hazards input assumption, not an empirical finding. The reference grid itself uses hypothetical multipliers defined against the same life-table baseline and therefore does not require transfer of an estimate from another cohort. Applying an HR reported in an external study is a separate step that assumes compatibility of the mortality endpoint, comparator, adjustment set, target population, and time pattern with the Japanese general-population baseline. Because HRs are non-collapsible and these elements often differ, an external HR should not be treated as a disease-versus-population multiplier without explicit justification [8,9,10].

### 2.4. Reference-Grid Analyses

We evaluated index ages of 65, 70, 75, 80, 85, and 90 years for men and women. The primary HR grid included 1.00, 1.25, 1.50, 2.00, 2.50, 3.00, and 4.00. These values were prespecified for interpretability and range coverage rather than selected by a formal frequency analysis of published HRs. An HR of 1.00 defined the life-table baseline; HRs of 1.25 and 1.50 represented modest increments; and HRs of 2.00–4.00 covered larger multipliers while keeping the table compact. Denser spacing near an HR of 1.00 provided additional resolution where the absolute change per HR increment was largest. For each age–sex–HR cell, we calculated median remaining lifespan, mean remaining years, and 1-, 3-, 5-, and 10-year survival. The 5-year horizon was selected as a compact intermediate illustration, not as a universally preferred decision horizon; all four horizons are supplied in machine-readable form.

To provide an age-based communication metric, we defined the baseline-equivalent age for each fixed-HR cell as the age on the sex-specific HR 1.00 curve with the same median remaining lifespan, using linear interpolation between adjacent attained ages. The baseline-equivalent age shift was the matched age minus the index age. This measure is related to prior effective-age and age-based translations of mortality ratios [14,15], but its matching rule is defined specifically by equality of median remaining lifespan on the population-average Japanese life-table baseline, rather than by matching an age-specific hazard or life-expectancy quantity. It is an actuarial comparison, not a biological age or patient-level prediction. To examine where approximate age invariance weakened, we fitted ordinary least-squares regressions of the natural logarithm of annual hazards derived from the one-year death probabilities on attained age over the age ranges of 65–89 and 100–110 years. The fitted Gompertz slopes were used only as shape diagnostics for the published life-table mortality curves and did not enter the translation calculations.

### 2.5. Illustrative Input-Uncertainty and Time-Pattern Analyses

To demonstrate propagation of input uncertainty, we transformed a hypothetical HR of 2.00 and its illustrative 95% confidence interval of 1.50–2.50. The two HR bounds were transformed separately, and the resulting output range was reported without treating it as a validated prediction interval. To examine departure from a constant HR, we compared an HR of 2.00 throughout with hypothetical sequences that applied an HR of 4.00 for the first 1 or 3 years and an HR of 2.00 thereafter, together with an HR of 4.00 throughout as a high constant-HR comparator. These inputs were selected only to make the effects of HR magnitude and duration visible. No scenario was assigned a disease label, linked to a published coefficient, or intended for comparison across clinical conditions. Definitions of the illustrative scenarios are provided in Supplementary Table S2.

### 2.6. Software and Reproducibility

All calculations were reproduced with Python 3.13.5 (Python Software Foundation, Beaverton, OR, USA), pandas 2.2.3 (NumFOCUS, Austin, TX, USA), and Matplotlib 3.10.8 (NumFOCUS, Austin, TX, USA). Code S1 contains the transcribed life-table probabilities and official remaining-life-expectancy values, the explicit terminal closure, transformation functions, agreement calculations, reference-grid analyses, baseline-equivalent age calculations, Gompertz slope diagnostics, sensitivity analyses, table generation, and figure generation. Data S1–S11 provide the baseline validation table, fixed-HR reference grids, time-pattern outputs, an external-HR applicability checklist, the full source data for Figures 3 and 4, and the baseline-equivalent age-shift grid.

## 3. Results

### 3.1. General-Population Life-Table Baseline

At an HR of 1.00, the computed mean remaining years reproduced the official complete-life-table remaining life expectancies. Across the 12 age–sex index cells, the mean absolute error was 0.004 years, the maximum absolute difference was 0.011 years, the mean absolute percentage error was 0.050%, and the maximum absolute percentage error was 0.230% (Table 1 and Data S1). Computed mean remaining years at the ages of 65, 70, 75, 80, 85, and 90 years were 20.0, 16.1, 12.5, 9.3, 6.6, and 4.5 years in men and 24.9, 20.5, 16.2, 12.2, 8.7, and 5.9 years in women. Median remaining lifespan exceeded the mean at ages of 65, 70, and 75 years in both sexes. At the age of 80 years, it was below the mean in men but remained above the mean in women; at ages of 85 and 90 years, it was below the mean in both sexes. The crossover therefore occurred between ages of 75 and 80 years in men and between ages of 80 and 85 years in women, reflecting later compression of the female survival distribution. Earlier deaths exert greater downward influence on the mean at the younger index ages, whereas a smaller group of longer survivors raises the mean relative to the median at older ages. At the age of 75 years, the baseline median remaining lifespan was 12.6 years in men and 16.8 years in women; the 5-year survival rates were 84.7% and 92.9%, respectively.

### 3.2. Reference Grid for Fixed Hazard Ratios

**Figure 1:**
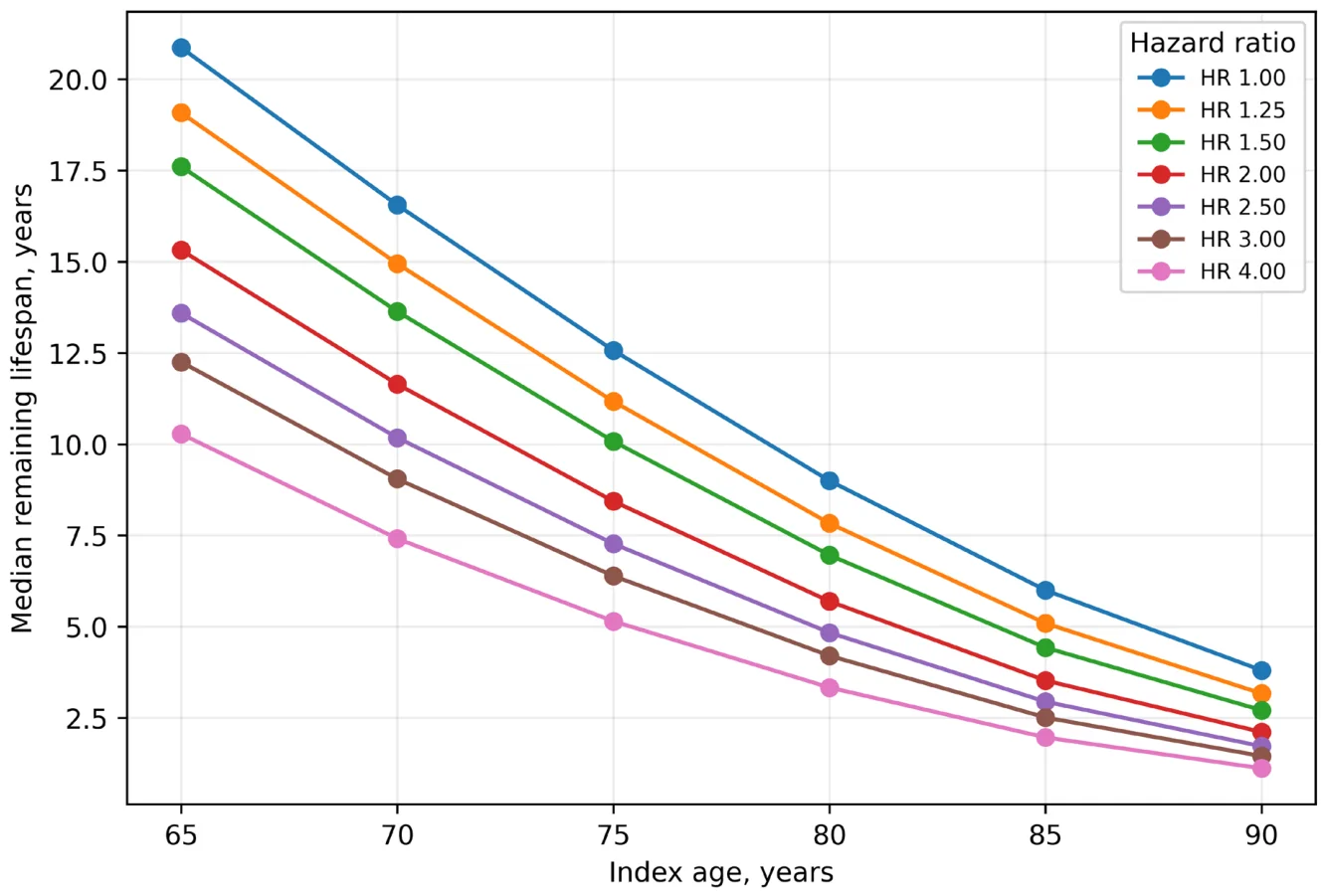
Median remaining lifespan by index age and hazard-ratio input in men. The curves show deterministic translations of fixed hazard ratio (HR) values using the Japanese male complete life table. An HR of 1.00 represents the general-population life-table baseline.

**Figure 2:**
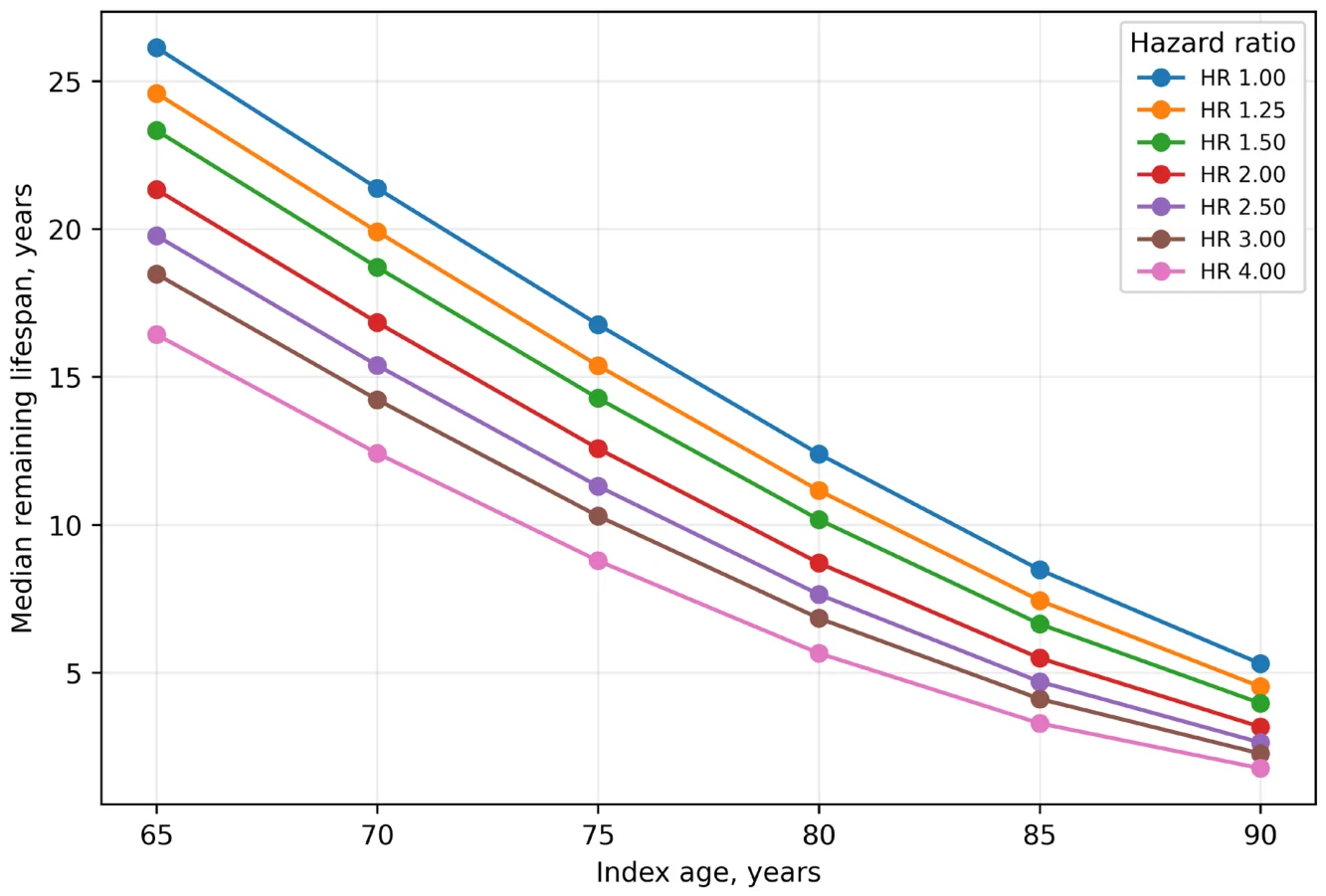
Median remaining lifespan by index age and hazard-ratio input in women. The curves show deterministic translations of fixed hazard ratio (HR) values using the Japanese female complete life table. An HR of 1.00 represents the general-population life-table baseline.

The HR grid showed a non-linear translation from relative hazard to remaining lifespan. In men aged 75 years, the median remaining lifespan decreased from 12.6 years at an HR of 1.00 to 11.2, 10.1, 8.4, 7.3, 6.4, and 5.1 years at HRs of 1.25, 1.50, 2.00, 2.50, 3.00, and 4.00, respectively (Table 2 and Figure 1). In women aged 75 years, the corresponding values were 16.8, 15.4, 14.3, 12.6, 11.3, 10.3, and 8.8 years (Table 2 and Figure 2). At the age of 75 years, successive increases from an HR of 1.00 to 1.50, from 1.50 to 2.00, and from 2.00 to 2.50 reduced the median by 2.49, 1.64, and 1.16 years in men and by 2.49, 1.70, and 1.27 years in women. These differences were computed at full precision; the one-decimal entries in Table 2 are rounded for display. Absolute separation also narrowed with advancing index age because the baseline survival time was shorter. For example, the difference between an HR of 1.00 and an HR of 4.00 was 10.6 years in men aged 65 years but 2.7 years in men aged 90 years.

Fixed-time survival probabilities provided a related but distinct view. At the age of 75 years in men, the 5-year survival was 84.7% at an HR of 1.00, 77.9% at an HR of 1.50, 71.7% at an HR of 2.00, 65.9% at an HR of 2.50, and 51.4% at an HR of 4.00. At the same age in women, the corresponding values were 92.9%, 89.5%, 86.3%, 83.2%, and 74.5%. The 5-year horizon is shown as an intermediate example rather than a universally preferred decision horizon. Results at 1, 3, 5, and 10 years are provided in Data S4–S6 so that users can select the horizon that matches the clinical question.

Matching each transformed median to the HR 1.00 curve expressed the same grid as a baseline-equivalent age shift (Table 3 and Data S11). For an HR of 2.00, the shift across index ages 65–90 years ranged from 5.6 to 6.7 years in men and from 4.7 to 5.0 years in women. At the age of 75 years, an HR of 2.00 corresponded to shifts of 5.8 years in men and 4.8 years in women. Thus, although the absolute years lost at an HR of 2.00 decreased with advancing age, the corresponding age-scale shift was comparatively stable. The values are actuarial matches to the population-average baseline and should not be interpreted as biological ages.

The age shift was least stable at the oldest index age and the highest HR. In men, the HR 4.00 shift was 11.4–13.1 years at index ages 65–85 years but 16.1 years at the age of 90 years, where the matched baseline age was 106.1 years. Approximate age invariance is expected when age-specific mortality is approximately log-linear [14,15]. In the Japanese male table, the fitted Gompertz slope declined from 0.106 per year at ages of 65–89 years to 0.066 per year at ages of 100–110 years. Matching at the oldest index age and highest HR therefore occurred where log-linearity weakened and should be interpreted cautiously.

### 3.3. Illustrative Input-Uncertainty and Time-Pattern Analyses

**Figure 3:**
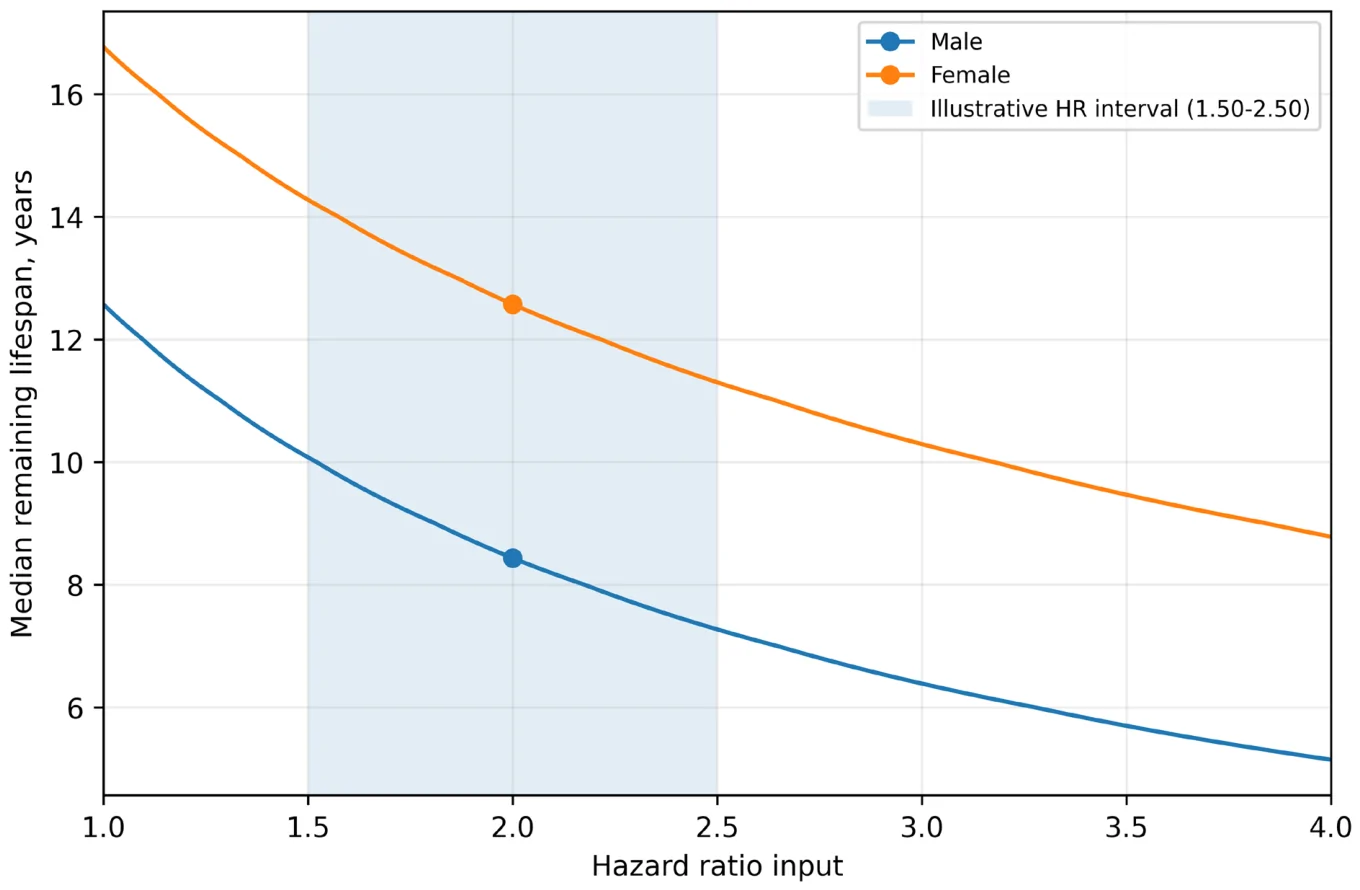
Non-linear translation and illustrative uncertainty propagation at an index age of 75 years. Curves show median remaining lifespan across hazard ratio (HR) inputs from 1.00 to 4.00 in men and women. Points mark an HR of 2.00, and the shaded band spans the hypothetical 95% confidence interval of 1.50–2.50. This input interval translates to median ranges of 7.3–10.1 years in men and 11.3–14.3 years in women. The shaded region is not a prediction interval.

For the hypothetical HR of 2.00 (95% confidence interval, 1.50–2.50), the point input at the age of 75 years translated to median remaining lifespans of 8.4 years in men and 12.6 years in women and to 5-year survival probabilities of 71.7% and 86.3%, respectively. Transforming the two HR bounds yielded median ranges of 7.3–10.1 years in men and 11.3–14.3 years in women; the corresponding 5-year survival ranges were 65.9–77.9% and 83.2–89.5% (Figure 3 and Data S9). These ranges represent propagation of uncertainty in the HR input only. They are not confidence intervals for a fitted prediction model and do not include uncertainty in the life-table baseline or model structure.

**Figure 4:**
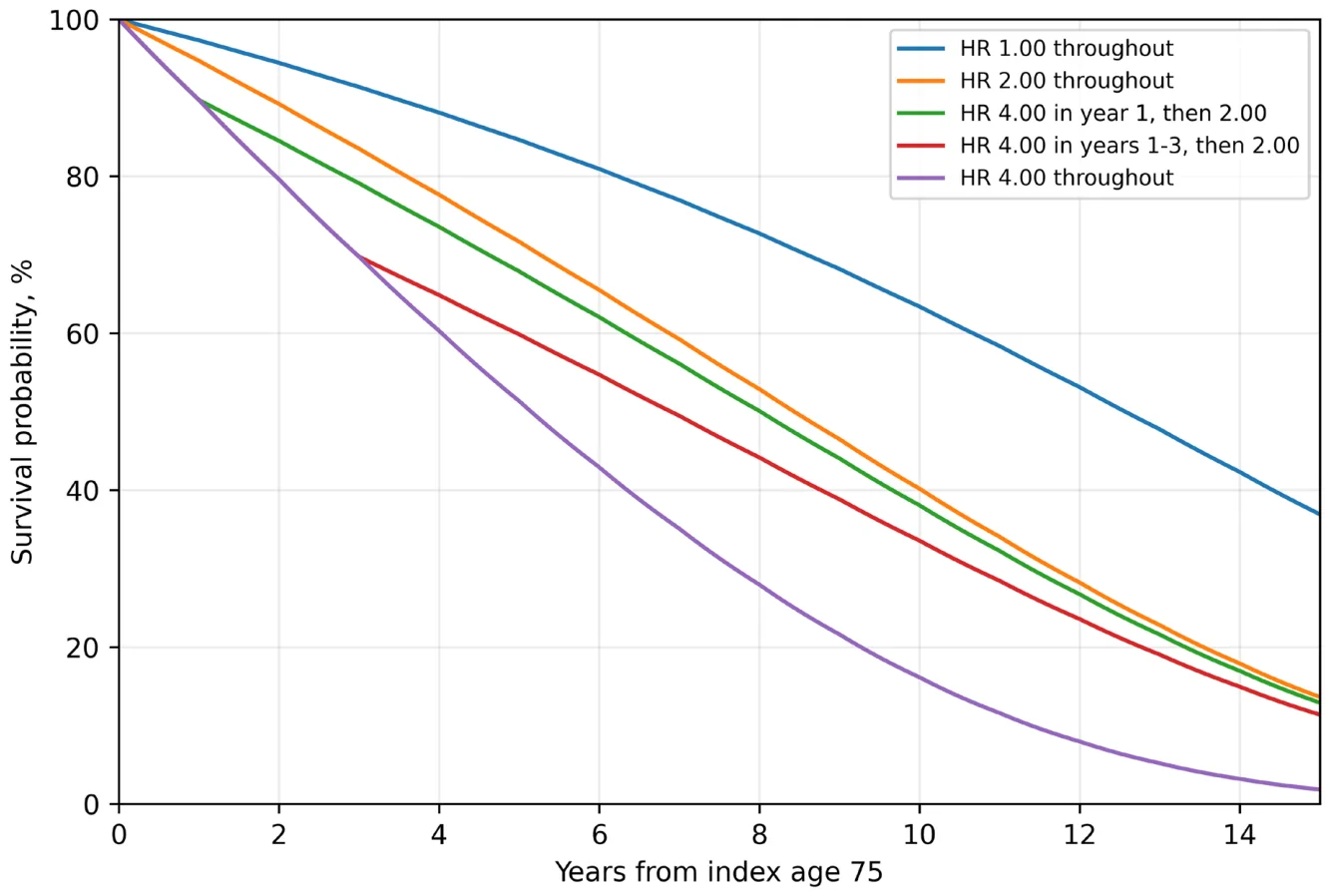
Sensitivity to the duration of an early elevated hazard at an index age of 75 years in men. Curves compare the life-table baseline, a hazard ratio (HR) of 2.00 throughout, an HR of 4.00 during the first year or first 3 years followed by an HR of 2.00, and an HR of 4.00 throughout. The sequences are purely hypothetical and show how departure from a constant-HR assumption changes the survival trajectory. The corresponding pattern was qualitatively similar in women; sex-specific values at the age of 75 years are reported in Table 4, and results across all index ages are provided in Data S7.

The time-pattern analysis showed that the chosen summary measure changes the apparent effect of front-loaded hazard (Table 4 and Figure 4). At the age of 75 years, an HR of 2.00 throughout produced median remaining lifespans of 8.44 years in men and 12.57 years in women and 5-year survival probabilities of 71.7% and 86.3%. Applying an HR of 4.00 for the first year and an HR of 2.00 thereafter reduced the median by only 0.42 years in men and 0.19 years in women, but reduced 5-year survival by 3.8 and 1.9 percentage points. Extending the HR to 4.00 through the first 3 years reduced the median by 1.54 and 0.67 years and the 5-year survival by 11.8 and 6.4 percentage points. The corresponding results at other index ages are provided in Data S7. These sequences are hypothetical sensitivity analyses, not estimates for a disease or treatment.

## 4. Discussion

Previous studies established the general principle of combining proportional mortality multipliers with life tables and of expressing mortality differences through life-expectancy, longevity, probability-based, or effective-age measures [12,13,14,15]. The present study therefore does not claim to provide the first HR-to-life-table transformation. Its contribution is to integrate three elements in one reproducible Japanese age- and sex-specific reference framework: a baseline-equivalent age shift matched on median remaining lifespan rather than on an age-specific hazard or life-expectancy quantity; joint reporting of median remaining lifespan, mean remaining years, fixed-time survival probabilities, and age shift in one grid; and a disease-neutral time-varying HR sensitivity analysis alongside the fixed-HR grid. The last element goes beyond a fixed-HR lookup framework by showing that median lifespan and fixed-horizon survival can respond differently to a front-loaded hazard. Agreement with official remaining life expectancy serves as an implementation check rather than an empirical finding.

The translation varied across index age, sex, HR magnitude, and outcome summary. Equal HR increments had progressively smaller effects at higher starting values. A given fixed HR removed more absolute years at younger index ages because baseline survival was longer; at older ages, it acted on a compressed remaining-lifetime distribution. The baseline-equivalent age shift offered a complementary view. For an HR of 2.00, absolute years lost decreased with age, whereas the age-scale shift remained approximately 5.6–6.7 years in men and 4.7–5.0 years in women. This stability may aid communication, but the matched age depends on the selected life table and summary metric and is not a biological-age estimate. The approximate invariance weakened at the age of 90 and an HR of 4.00, particularly in men, because the matched age entered the late-life mortality-deceleration region rather than the approximately log-linear adult range. Fixed-time survival remains preferable when a decision depends on reaching a specific horizon, which should be chosen for the decision rather than assumed to be 5 years in every setting.

The distinction between a general-population baseline and an unexposed baseline is essential. An HR of 1.00 represents the population-average mortality experience in the Japanese complete life table and is not a healthy reference profile. The fixed-HR grid is internally self-consistent because each multiplier is defined against that same baseline. Mapping an HR from an external study is a separate analytical step. An HR estimated against a selected clinical subgroup, a covariate-defined reference category, or another active treatment should not be treated as a disease-versus-general-population multiplier without justification. Compatibility of the endpoint, comparator, adjustment set, target population, and follow-up pattern must be considered, particularly because HRs are non-collapsible [8,9,10]. The applicability checklist in Supplementary Table S3 and Data S8 makes these requirements explicit.

Because the transformation operates only on the supplied HR sequence and the baseline survival curve, two inputs with the same HR sequence produce identical outputs regardless of any label attached to them. A condition name therefore carries no numerical information in this framework unless it changes the HR magnitude, its trajectory over time, or the baseline survival source. This is a mathematical property of the translation and is the reason the reference grid and sensitivity analyses are presented without clinical labels; it does not justify transferring an externally estimated HR to an incompatible baseline.

The uncertainty analysis is deliberately limited to deterministic propagation of the HR bounds. It excludes uncertainty in the source-study baseline, sampling variation in the life table, model misspecification, and covariance between parameters. The time-pattern analysis adds a separate interpretive point: a short front-loaded hazard changed the median much less than it changed 5-year survival. Median remaining lifespan can therefore understate an early risk concentration even when it summarizes the full survival trajectory. Reporting both a lifespan measure and decision-relevant fixed-time survival is particularly important when proportional hazards are doubtful. Source-specific time-varying effects or baseline survival curves remain preferable when available.

Several limitations arise directly from the design. First, the framework is not an internally or externally validated prediction model; no participant-level outcomes were available to estimate calibration, discrimination, or prediction error. Second, agreement with official remaining life expectancy confirms numerical implementation at an HR of 1.00 but does not establish clinical validity at HR values above 1.00. Third, the fixed-HR grid assumes that the same multiplier applies at each attained age over the remaining-life trajectory. The hypothetical time-varying analyses illustrate sensitivity to this assumption but do not identify the correct pattern for any condition. Fourth, application of an external HR requires transportability assumptions that may fail because of differences in estimand, comparator, covariate adjustment, endpoint, geography, calendar period, and care setting. Fifth, HRs do not generally have a simple causal interpretation, and mathematical translation does not remove non-collapsibility or selection over follow-up [8,9,10]. Sixth, the baseline-equivalent age shift is specific to the chosen median-based matching rule and should not be interpreted as biological ageing; its approximate age invariance also weakens at the oldest index ages, where late-life mortality deceleration departs from log-linearity and matched ages lie in sparsely surviving tails. Finally, the 2020 Japanese complete life table may not represent other countries, later calendar periods, or selected clinical populations, and an HR of 1.00 already includes chronic disease and frailty at their population frequencies. At the time of this revision (15 August 2026), the 23rd Complete Life Tables remained the most recent complete life tables released by the Ministry of Health, Labour and Welfare. The 2025 abridged life tables had been released, and the Ministry indicated that a complete life table based on the 2025 census was planned but had not yet been released [16,17]. Because abridged and complete life tables use different population and vital-statistics sources, we retained the latest complete life table to preserve consistency with the prespecified design. Updating the baseline would require recalculation of all absolute grid values because they depend on age- and sex-specific death probabilities. The transformation algorithm and qualitative ordering across HR inputs would remain unchanged, but the magnitude of each estimate should be recomputed rather than assumed.

Within these boundaries, the framework has practical value as a mathematical interpretation and communication aid. A reader can inspect the age–sex grid, translate the endpoints of an HR confidence interval, express a fixed-HR result on a baseline-equivalent age scale, and test alternative time patterns. Before applying a published HR, the source estimand and comparator should be checked against the conditions in Supplementary Table S3. Results from HRs estimated under different comparators or models should not be compared as if they represented disease severity on one common scale. The translated quantities may support discussion of time horizons, but they should not be converted into automatic treatment rules without source-specific evidence, patient preferences, and clinical validation.

## 5. Conclusions

A life-table transformation can translate specified mortality HR inputs into median remaining lifespan, mean remaining years, fixed-time survival probabilities, and baseline-equivalent age shifts in Japanese older adults. The grid makes non-linearity and age-dependent absolute effects visible, while the time-pattern analysis shows that median lifespan and fixed-horizon survival can respond differently to front-loaded hazard. Applying a published HR requires separate judgments about proportionality, comparator alignment, and transportability. The output is therefore a reproducible reference resource, not a validated patient-specific or disease-specific prognostic model.

## Figures and Tables

**Table 1:** General-population life-table baseline metrics by age and sex.

Sex	Age, Years	Median, Years	Computed Mean, Years	1-Year Survival, %	3-Year Survival, %	5-Year Survival, %	10-Year Survival, %
Male	65	20.9	20.0	99.0	96.7	93.9	84.7
Male	70	16.6	16.1	98.3	94.6	90.2	76.4
Male	75	12.6	12.5	97.3	91.4	84.7	63.4
Male	80	9.0	9.3	95.6	85.9	74.9	43.6
Male	85	6.0	6.6	92.1	75.3	58.3	21.9
Male	90	3.8	4.5	85.8	59.5	37.5	7.5
Female	65	26.1	24.9	99.6	98.6	97.5	93.4
Female	70	21.4	20.5	99.3	97.8	95.9	89.1
Female	75	16.8	16.2	98.9	96.2	92.9	80.4
Female	80	12.4	12.2	97.9	92.9	86.5	64.0
Female	85	8.5	8.7	95.7	85.8	74.0	39.2
Female	90	5.3	5.9	91.2	72.4	53.0	15.5

Note: HR, hazard ratio. An HR of 1.00 is the Japanese general-population complete-life-table baseline, not a disease-free baseline. Computed mean remaining years were obtained by trapezoidal integration of the annual survival curve. Across the 12 age–sex cells, the mean absolute error versus official remaining life expectancy was 0.004 years and the maximum absolute difference was 0.011 years; the comparison uses published source values reported to finite precision.

**Table 2:** Median remaining lifespan in years by age, sex, and hazard-ratio input.

Sex	Age, Years	HR 1.00	HR 1.25	HR 1.50	HR 2.00	HR 2.50	HR 3.00	HR 4.00
Male	65	20.9	19.1	17.6	15.3	13.6	12.3	10.3
Male	70	16.6	14.9	13.6	11.6	10.2	9.0	7.4
Male	75	12.6	11.2	10.1	8.4	7.3	6.4	5.1
Male	80	9.0	7.8	7.0	5.7	4.8	4.2	3.3
Male	85	6.0	5.1	4.4	3.5	2.9	2.5	2.0
Male	90	3.8	3.2	2.7	2.1	1.7	1.5	1.1
Female	65	26.1	24.6	23.3	21.3	19.8	18.5	16.4
Female	70	21.4	19.9	18.7	16.8	15.4	14.2	12.4
Female	75	16.8	15.4	14.3	12.6	11.3	10.3	8.8
Female	80	12.4	11.2	10.2	8.7	7.6	6.8	5.7
Female	85	8.5	7.4	6.6	5.5	4.7	4.1	3.3
Female	90	5.3	4.5	4.0	3.2	2.6	2.3	1.8

Note: HR, hazard ratio. Values are deterministic life-table translations and are rounded to one decimal place.

**Table 3:** Baseline-equivalent age shift in years by age, sex, and hazard-ratio input.

Sex	Age, Years	HR 1.00	HR 1.25	HR 1.50	HR 2.00	HR 2.50	HR 3.00	HR 4.00
Male	65	0.0	2.0	3.7	6.5	8.7	10.4	13.1
Male	70	0.0	2.0	3.6	6.2	8.3	9.9	12.5
Male	75	0.0	1.9	3.4	5.8	7.7	9.3	11.7
Male	80	0.0	1.8	3.2	5.6	7.4	8.9	11.4
Male	85	0.0	1.8	3.4	5.8	7.7	9.5	12.7
Male	90	0.0	1.9	3.6	6.7	9.5	11.9	16.1
Female	65	0.0	1.6	2.9	5.0	6.7	8.1	10.4
Female	70	0.0	1.6	2.9	4.9	6.6	7.9	10.0
Female	75	0.0	1.6	2.8	4.8	6.3	7.6	9.6
Female	80	0.0	1.5	2.8	4.7	6.2	7.4	9.4
Female	85	0.0	1.5	2.7	4.7	6.2	7.4	9.6
Female	90	0.0	1.5	2.8	4.9	6.8	8.5	11.3

Note: HR, hazard ratio. Each value is the age on the sex-specific HR 1.00 median curve that matches the transformed median, minus the index age. Matching used linear interpolation between adjacent attained ages. An HR of 1.00 therefore has a shift of 0.0 years. These are actuarial comparisons to the population-average baseline, not biological ages. At the oldest index age and highest HR, the matched age enters the late-life tail where log mortality is less linear; those cells should be interpreted cautiously.

**Table 4:** Sensitivity to hypothetical hazard-ratio time patterns at an index age of 75 years.

Scenario	HR Input	Male Median, Years (Change)	Female Median, Years (Change)	Male 5-Year Survival, % (Change)	Female 5-Year Survival, % (Change)
HR of 2.00 throughout	2.00 throughout	8.44 (reference)	12.57 (reference)	71.7 (reference)	86.3 (reference)
Early elevation for 1 year	4.00 in year 1; 2.00 thereafter	8.01 (−0.42)	12.38 (−0.19)	67.9 (−3.8)	84.4 (−1.9)
Early elevation for 3 years	4.00 in years 1–3; 2.00 thereafter	6.90 (−1.54)	11.91 (−0.67)	59.9 (−11.8)	79.9 (−6.4)
HR of 4.00 throughout	4.00 throughout	5.15 (−3.29)	8.78 (−3.79)	51.4 (−20.3)	74.5 (−11.8)

Note: HR, hazard ratio. Values in parentheses are changes from an HR of 2.00 throughout; median changes are in years and survival changes are in percentage points. The sequences are disease-neutral sensitivity analyses and are not disease- or treatment-specific estimates.

## Data Availability

The source life-table data are publicly available from the Ministry of Health, Labour and Welfare of Japan [16] at https://www.mhlw.go.jp/toukei/saikin/hw/life/23th/index.html, accessed on 23 August 2026. All derived data, the external-HR applicability checklist, and the complete analysis code supporting the reported results are included in the Supplementary Materials (Code S1 and Data S1–S11).

## Supplementary Materials

The following supporting information can be downloaded at https://www.mdpi.com/article/10.3390/geriatrics11050116/s1, Table S1, Supplementary files supplied with the manuscript; Table S2, Definitions of disease-neutral illustrative HR scenarios; Table S3, Checklist for assessing whether an external hazard ratio can be applied to the life-table baseline; Code S1, Python source code; Data S1, Baseline life-table validation metrics; Data S2, Median remaining-lifespan reference grid; Data S3, time-pattern scenarios at the age of 75 years; Data S4, Full HR-grid metrics; Data S5, Five-year survival reference grid; Data S6, Ten-year survival reference grid; Data S7, time-pattern scenarios across all six index ages; Data S8, Machine-readable external-HR applicability checklist; Data S9, Fine-grid uncertainty-propagation and Figure 3 source data; Data S10, Source data for the time-pattern survival curves; and Data S11, Baseline-equivalent age shifts.
