# Supplementary material for: Translating Mortality Hazard Ratios into Remaining-Lifespan Measures in Older Adults Using Japanese Complete Life Tables: A Deterministic Reference-Table Study
Source: Geriatrics (Basel). 2026 Aug 31;11(5):116. doi: 10.3390/geriatrics11050116 (PMC13600169; doi:10.3390/geriatrics11050116)
Supplement: Supplementary file 1 [file geriatrics-11-00116-s001.zip › Supplementary_Material_Revised.pdf]

Supplementary Materials

Translating Mortality Hazard Ratios into Remaining-Lifespan Measures in Older Adults Using Japanese Complete Life Tables: A Deterministic Reference-Table Study

The main analysis is the age-sex-hazard-ratio (HR) reference grid derived from the Japanese complete life tables. The revised supplementary analyses are disease-neutral and purely hypothetical. They show how an HR input interval propagates to remaining-lifespan outputs, how the duration of an early elevated hazard changes the survival trajectory, and how each fixed-HR result maps to a baseline-equivalent age shift. Code S1 also reproduces the late-life Gompertz slope diagnostic used to identify where approximate age invariance of the age shift weakens. Table S3 provides an applicability checklist for external HRs. When the listed conditions are not sufficiently met, the result should be described as a mathematical scenario rather than a transported or validated prognosis.

Table S1. Supplementary files supplied with the manuscript.

Item	File name	Format	Content
Supplementary Material	Supplementary_Material_Revised.docx	.docx	This document, including Tables S1-S3.
Code S1	Supplementary_Code_S1.py	.py	Python source code for all calculations, CSV outputs, figures, and late-life Gompertz slope diagnostics.
Data S1	Supplementary_Data_S1_Baseline_Life_Table_Metrics.csv	.csv	Baseline metrics, official remaining life expectancy, and cell-level agreement.
Data S2	Supplementary_Data_S2_Median_Remaining_Lifespan_Reference.csv	.csv	Median remaining lifespan by age, sex, and fixed-HR input.
Data S3	Supplementary_Data_S3_Time_Pattern_Scenarios_Age75.csv	.csv	Time-pattern scenarios and changes from HR 2.00 at age 75 years.
Data S4	Supplementary_Data_S4_Full_HR_Grid_Metrics.csv	.csv	All calculated metrics for each age-sex-HR reference-grid cell.
Data S5	Supplementary_Data_S5_Five_Year_Survival_Reference.csv	.csv	Five-year survival probability by age, sex, and fixed-HR input.
Data S6	Supplementary_Data_S6_Ten_Year_Survival_Reference.csv	.csv	Ten-year survival probability by age, sex, and fixed-HR input.
Data S7	Supplementary_Data_S7_Time_Pattern_Scenarios_All_Ages.csv	.csv	Time-pattern scenario outputs across all index ages and both sexes.
Data S8	Supplementary_Data_S8_External_HR_Applicability_Checklist.csv	.csv	Machine-readable external-HR applicability checklist.
Data S9	Supplementary_Data_S9_Uncertainty_Propagation_and_Figure3_Source.csv	.csv	Fine HR grid for both sexes and all index ages, including Figure 3 curve data and interval markers.
Data S10	Supplementary_Data_S10_Time_Pattern_Survival_Curves.csv	.csv	Source data for the time-pattern survival curves shown in Figure 4.
Data S11	Supplementary_Data_S11_Baseline_Equivalent_Age_Shifts.csv	.csv	Baseline-equivalent ages and age shifts for all fixed-HR reference-grid cells.

Table S2. Definitions of disease-neutral illustrative HR scenarios.

Scenario group	Scenario	HR input	Pattern	Purpose and interpretation
Reference	Life-table baseline	1.00 throughout	Constant	Japanese general-population life-table baseline; not a disease-free baseline.
Input uncertainty	Illustrative lower HR input	1.50 throughout	Constant	Hypothetical lower limit used to demonstrate propagation of an HR input interval.
Input uncertainty and time-pattern reference	Central/reference HR input	2.00 throughout	Constant	Hypothetical point input for uncertainty propagation and reference pattern for time-pattern changes.
Input uncertainty	Illustrative upper HR input	2.50 throughout	Constant	Hypothetical upper limit used to demonstrate propagation of an HR input interval.
Time pattern	Early elevation for 1 year	4.00 in year 1; 2.00 thereafter	Time-dependent	Tests one year of higher early hazard before the HR decreases to 2.00.
Time pattern	Early elevation for 3 years	4.00 in years 1-3; 2.00 thereafter	Time-dependent	Tests three years of higher early hazard before the HR decreases to 2.00.
Time pattern	Constant high HR comparator	4.00 throughout	Constant	High constant-HR comparator for the time-pattern sensitivity analysis.

Note: HR, hazard ratio. The inputs in Table S2 were selected only to demonstrate deterministic input-interval and time-pattern sensitivity. They were not derived from a clinical cohort and should not be interpreted as disease- or treatment-specific coefficients. Two inputs with the same HR sequence produce identical outputs regardless of the label attached to them.

Table S3. Checklist for assessing whether an external hazard ratio can be applied to the life-table baseline.

Domain	Question	Why it matters	Recommended interpretation or action
Endpoint	Does the external HR describe all-cause mortality?	The life table models all-cause mortality. Cause-specific or composite endpoints require additional competing-risk or multistate modeling.	Use an all-cause mortality HR, or describe the calculation only as a hypothetical multiplier scenario.
Estimand	Is the HR a population-risk contrast rather than a treatment-effect contrast?	An HR comparing two active treatments estimates a different contrast from disease or exposure versus a general-population baseline.	Do not relabel a treatment-effect HR as a disease-versus-population HR.
Comparator and baseline	Does the source comparator approximate the life-table reference population?	An HR estimated against a selected clinical subgroup is not automatically transportable to the national general-population hazard.	Require a sufficiently aligned comparator or state that the output is a scenario, not a transported prognosis.
Target population	Are age, sex, geography, calendar period, and care setting sufficiently aligned?	Absolute survival depends on baseline risk, which changes across populations and periods.	Use the closest available life table or recalibrate with a source-population baseline survival curve.
Adjustment and non-collapsibility	Are the source adjustment set and estimand appropriate for the intended interpretation?	Adjusted and unadjusted HRs can differ even without confounding because HRs are non-collapsible.	Preserve the source-study interpretation and avoid comparisons across HRs derived under different models.
Time pattern	Is proportional hazards plausible over the projected horizon?	Applying one HR over 10-25 years assumes a constant multiplier at every attained age.	Use time-varying HR inputs or restrict the projection horizon when proportionality is not plausible.
Uncertainty	Are confidence limits or another plausible HR range available?	A single deterministic output conceals uncertainty in the externally estimated HR.	Translate lower and upper HR inputs and report the resulting output range without calling it a validated prediction interval.
Validation	Has the translated output been calibrated and externally validated for the intended population?	Mathematical consistency does not establish patient-level calibration or discrimination.	Use the grid as an interpretive reference unless validation against participant-level outcomes has been performed.

Note: HR, hazard ratio. The checklist makes explicit the conditions that must be considered before an externally reported HR is applied to a general-population life-table baseline. Failure in one or more domains does not alter the arithmetic of the transformation, but it limits the result to a hypothetical scenario.
